# A Case of Rheumatic Fever Treated with a Cold Bath; Death Occurring Immediately on Leaving the Bath

**Published:** 1874-01

**Authors:** Sydney Ringer


*A Case of Rheumatic Fever Treated with a Cold Bath; Death occurring immediately on leaving the Bath.* By SYDNEY RINGER, M.D.

These short notes are published, as this case will help to answer the following questions: Can cold baths be administered in rheumatic fever without danger? and is it advisable before employing this treatment to wait for the onset of hyperpyrexia? or should we commence it when high fever, absence of joint pain, suppression of perspiration, and delirium, show that there is danger that hyperpyrexia may occur? As hitherto all cases of rheumatic hyperpyrexia have proved fatal unless treated by cold baths, it is obvious that this case in no way contra-indicates that treatment on the occurrence of this dangerous condition.

A young woman, aged 24, was admitted into University College Hospital with rheumatic fever. Her father died suddenly from some unknown cause. Four years before, the patient suffered from a severe attack of rheumatic fever. Her present illness begun about a week before her admission into hospital. On her admission she suffered from a sharp attack of rheumatic fever; her temperature rising daily to 103°. There was not, however, much joint affection, and at first she perspired freely, but latterly her skin grew dry. She rapidly got worse: thus during the nine days she was in hospital her temperature rose daily till it reached 105°, and her respirations rose from 32 to 60; her pulse remained about 120 per minute, and throughout was strong. Latterly she suffered from dyspnœa, and subsequently was propped up in bed with pillows. She wandered a little at night, and on the day the bath was employed her intellect was a little obscured, and she passed her urine under her. At 7.42 P. M. of the ninth day of her admission she was placed in a general bath of 92°, her temperature in the axilla being 105° Fahr. In seven minutes, and before the temperature of the bath was reduced, her rectal temperature was 105.8°; the temperature of the bath was then reduced. In eighteen minutes after the commencement of the bath her rectal temperature was 105.4°. After forty-four minutes her temperature had fallen to 103.4°, the temperature of the bath being 69°. Whilst in the bath she took 4 ozs. of brandy. She was removed because her breathing grew rather shallow. After being put to bed she merely gasped a few times for five minutes and died, notwithstanding the employment of artificial respiration, energetic friction to the surface of the body, and anal injections of brandy. At the post-mortem examination we found a few patches of recent lymph on both lungs, but not an unnatural quantity of serosity in the pleuræ. The heart was universally adherent to the pericardium, the adhesions being tough; the blood in the heart and great vessels was very dark-colored fluid and free from clots. The left ventricle and auricle were dilated—especially the auricle. On the tricuspid, mitral, and aortic valves, numerous minute vegetations were seen at the usual places. The mitral, aortic, and pulmonary valves were a good deal thickened. The mitral valves admitted three fingers nearly to the knuckles; the two segments were united for a short distance; they permitted some regurgitation when tested at the tap. The heart's substance looked healthy, and was of fair consistence. On the surface, at places, there was a thin line of paler and rather opaque tissue. The walls of the left ventricle at the base were half an inch thick, at the middle five-eighths of an inch, and at the apex rather less than quarter of an inch. The brain, liver, spleen, kidneys, stomach, and intestines, were healthy. During life her urine contained a trace of albumen.—*The Practitioner.*

